# The relationship between prenatal exposure to organophosphate insecticides and neurodevelopmental integrity of infants at 5-weeks of age

**DOI:** 10.3389/fepid.2022.1039922

**Published:** 2022-12-14

**Authors:** Supattra Sittiwang, Pimjuta Nimmapirat, Panrapee Suttiwan, Wathoosiri Promduang, Nattapon Chaikittipornlert, Trecia Wouldes, Tippawan Prapamontol, Warangkana Naksen, Nattawadee Promkam, Sureewan Pingwong, Adrian Breckheimer, Valerie Cadorett, Parinya Panuwet, Dana Boyd Barr, Brittney O. Baumert, Pamela Ohman-Strickland, Nancy Fiedler

**Affiliations:** 1 LIFE Di Center, Faculty of Psychology, Chulalongkorn University, Bangkok, Thailand; 2 Department of Psychological Medicine, The University of Auckland, Auckland, New Zealand; 3 Research Institute for Health Sciences, Chiang Mai University, Chiang Mai, Thailand; 4 Faculty of Public Health, Chiang Mai University, Chiang Mai, Thailand; 5 School of Public Health, Rutgers University, Piscataway, NJ, United States; 6 Gangarosa Department of Environmental Health, Rollins School of Public Health, Emory University, Atlanta, GA, United States; 7 Department of Population and Public Health Sciences, Keck School of Medicine, University of Southern California, Los Angeles, CA, United States; 8 Environmental and Occupational Health Science Institute, Rutgers University, Piscataway, NJ, United States

**Keywords:** organophosphates, NICU network neurobehavioral scale (NNNS), newborn, Thailand, farming, insecticides

## Abstract

**Introduction:**

Organophosphate (OP) insecticides are among the most abundantly used insecticides worldwide. Thailand ranked third among 15 Asian countries in its use of pesticides per unit hectare and fourth in annual pesticide use. More than 40% of Thai women of childbearing age work on farms where pesticides are applied. Thus, the potential for pregnant women and their fetuses to be exposed to pesticides is significant. This study investigated the relationship between early, mid, and late pregnancy maternal urine concentrations of OP metabolites and infant neural integrity at 5 weeks of age.

**Method:**

We enrolled women employed on farms from two antenatal clinics in the Chiang Mai province of northern Thailand. We collected urine samples monthly during pregnancy, composited them by early, mid and late pregnancy and analyzed the composited samples for dialkylphosphate (DAP) metabolites of OP insecticides. At 5 weeks after birth, nurses certified in use of the NICU Network Neurobehavioral Scale (NNNS) completed the evaluation of 320 healthy infants. We employed generalized linear regression, logistic and Poisson models to determine the association between NNNS outcomes and DAP concentrations. All analyses were adjusted for confounders and included creatinine as an independent variable.

**Results:**

We did not observe trimester specific associations between DAP concentrations and NNNS outcomes. Instead, we observed statistically significant inverse associations between NNNS arousal (β = −0.10; CI: −0.17, −0.002; *p* = 0.0091) and excitability [0.79 (0.68, 0.92; *p* = 0.0026)] among participants with higher average prenatal DAP concentrations across pregnancy. We identified 3 NNNS profiles by latent profile analysis. Higher prenatal maternal DAP concentrations were associated with higher odds of being classified in a profile indicative of greater self-regulation and attention, but arousal and excitability scores below the 50th percentile relative to US normative samples [OR = 1.47 (CI: 1.05, 2.06; *p* = 0.03)]. Similar findings are also observed among infants with prenatal exposure to substances of abuse (e.g., methamphetamine).

**Discussion:**

Overall, the associations between prenatal DAP concentrations and NNNS summary scores were not significant. Further evaluations are warranted to determine the implications of low arousal and excitability for neurodevelopmental outcomes of attention and memory and whether these results are transitory or imply inadequate responsivity to stimulation among children as they develop.

## Introduction

Organophosphate (OP) insecticides are among the most abundantly used insecticides worldwide that tend to be used in greater amounts and with fewer restrictions in low- and middle-income countries (LMICs) despite their well-documented acute neurotoxic effects (1). Globally, Thailand is one of the top countries in agricultural pesticide use (2). The country continues to increase its annual import of pesticides for agriculture with OP insecticides being listed among the most imported insecticide used to control pests for crops such as vegetables, rice, and flowers (3). Women in their childbearing years comprise ~40% of the workforce on Thai farms (4). Thus, for those women working on farms where pesticides are used, the potential for their fetus to be exposed to pesticides during the prenatal period is significant (5).

The primary mode of OP insecticide acute toxicity is by binding to acetylcholinesterase (AChE) thus preventing the breakdown of the neurotransmitter acetylcholine. Other non-cholinergic effects on the nervous system such as altered neuronal proliferation and impaired cognition have also been reported after low level exposures to OP insecticides. Previous human and animal studies have reported that neurotoxic insecticides cross the placental barrier and enter into the fetal blood stream (6, 7). Because the fetal detoxification process is immature, insecticide exposures during critical windows of fetal development produce lasting disruption of neurulation, neuron proliferation, neural migration, myelination, and synaptogenesis with specific sensitivity of the serotonin and dopamine systems as reported in animal models (8–10).

Although inconsistencies exist, epidemiologic studies from four US birth cohorts show that early indicators of growth and later cognitive development are adversely affected by prenatal insecticide exposure even without evidence of AChE inhibition during pregnancy (11–20). For example, lower birth weight and length, and shorter gestation were linked to maternal OP exposure (16, 19, 21) and, of note, infants of mothers whose paraoxonase status dictated slow OP insecticide metabolism exhibited smaller head circumference (20). In addition, studies document that increased concentrations of OP exposure *in utero* result in reduced fetal length and weight estimated from ultrasound at 20 weeks gestation. Neurodevelopmental deficits in neonates such as abnormal reflexes have also been noted in several studies (14, 22–24). A birth cohort study in China reported that high exposure of pregnant women to OP insecticides was the predominant risk factor for a lower summary score on the Neonatal Behavioral Neurologic Assessment (25). More recently, results from a cohort study of prenatal exposure to multiple pesticides in Chinese infants showed that auditory brainstem response (ABR) processing was slower in infants with greater prenatal pesticide exposure, indicating impaired neuromaturation (26). In addition to the effects on birth outcomes and neural integrity in the first few weeks of life, total diethylalkylphosphate (DEAP) and total dialkylphosphate (DAP) metabolite concentrations, measured at 28 weeks gestation, were significantly associated with reduced motor composite scores on the Bayley Scales of Infant Development III administered at 5 months (27).

Although several birth cohort studies investigating the associations of OP insecticide exposure on birth outcomes and neurodevelopment have been conducted, characterization of exposure in each trimester of pregnancy has been difficult to accomplish. For example, previous birth cohort studies relied upon a single measurement of OP exposure throughout the pregnancy (22, 28). In addition, with one exception, previous studies used the Brazelton Neonatal Behavioral Assessment Scale (BNBAS) to assess infant neural integrity (29). Although widely used, BNBAS examiners are trained to elicit optimal performance of the infant rather than a more objective assessment of the infant’s ability to organize their own behavior. Additionally, norms are not available for the BNBAS partly due to variability among examiners in seeking optimal infant performance (30). Of note, one study using the NICU Network Neurobehavioral Scale (NNNS) to evaluate the effect of OP insecticides on neurological development reported no detrimental effects of gestational exposure to OP insecticides on neurobehavioral outcomes among 5-week old infants. Rather, the study found that higher creatinine-corrected urinary concentrations of diethylphosphate metabolite were associated with improved attention, reduced lethargy and hypotonia among infants while higher creatinine-corrected urinary concentrations of total DAP metabolites were associated with fewer signs of autonomic stress (31). However, this study obtained spot urine samples at two-points during 3 trimesters of pregnancy which could contribute to exposure misclassification (31, 32).

The aims of the present study were to investigate the associations of OP insecticide exposure during each trimester of pregnancy and summed across pregnancy on the neural integrity of Thai infants. Our study was intended to address gaps in the existing literature by capturing OP metabolites during early, mid and late pregnancy and employing an objective measure of infant neural integrity at 5 weeks of age after resolution of birth trauma. Determining specific windows of vulnerability during gestation could inform prevention measures to reduce immediate and life-long adverse outcomes.

## Methods

### Participants and recruitment

The SAWASDEE study is a prospective birth cohort designed to examine the impact of prenatal, occupational exposure to OP insecticides on infant neurodevelopment. Between July 30 and June 30, 1,290 pregnant women who presented at Chom Thong or Muang Chum Tambon (Fang) hospitals, in Chiang Mai province, Thailand for their first antenatal care were screened by trained nurses. A total of 394 pregnant women who met the inclusion criteria listed in Table 1 were recruited for the study. Informed consent was obtained from all study participants prior to enrollment. This study was reviewed and approved by the Institutional Review Board at Emory University (with Rutgers University reliance) and the Ethical Review Board at Chiang Mai University (with Chulalongkorn University reliance). We excluded 61 pregnant women for the following reasons: miscarriage (*n* = 21), blighted ovum (*n* = 7), no longer receiving care at participating clinics (*n* = 11), illness (e.g., diabetes, thyroid, hepatitis) (*n* = 6), loss to follow-up or drop-out (*n* = 6), pregnancy complications (e.g., ectopic, fetal abnormality) (*n* = 4), twin pregnancy (*n* = 3), GA >20 weeks by ultrasound (*n* = 2), and substance abuse (*n* = 1).

A total of 333 pregnant women delivered live infants. Infants with the following conditions were excluded: GA < 32 weeks (*n* = 1), birth weight < 30 gm (*n* = 2), birth defect (i.e., prefrontal brain damage, meningomyelocele, congenital heart disease; *n* = 3), significant developmental delay (i.e., motor developmental delay at 6 months), hydrocephalus (*n* = 2), loss to follow-up (*n* = 1), and moved (*n* = 1). Three eligible participants did not complete the NNNS but remained in the study for subsequent neurodevelopmental tests. The final sample of 320, healthy 5-week old infants were administered the NNNS at the SAWASDEE clinic.

### Interview, medical record abstraction, and questionnaires

Trained, registered nurses recruited pregnant women and administered an intake questionnaire to determine study eligibility in community and district hospitals. After informed consent, trained research assistants administered a structured questionnaire to collect the following demographic, medical, and occupational information: age, education, marital status, ethnicity, primary language (Thai/non-Thai), parity/gravidity, monthly income, work history, pesticide use, drug and alcohol use, smoking history (mother and father), acute and chronic medical diagnoses, medication use, vitamins, and stressors (e.g., abuse, disasters, death in family). This questionnaire was repeated at mid and late pregnancy to determine changes in medical or occupational information. Maternal weight gain as a surrogate for nutritional status was based on the change in weight from the intake visit during the first trimester to mother’s weight at delivery. Because it is difficult to determine income among farm workers in LMIC settings, we developed an Observation of Assets Questionnaire (assets questionnaire) as an alternate measure of income (33, 34). Participants completed the assets questionnaire which was factor analyzed, yielding 3 primary factors: (1) Housing Quality: items rated to reflect quality of building materials and utilities (e.g., electricity, flooring); (2) Safety of home and neighborhood: participant perceptual ratings of home and neighborhood, and (3) Asset value: total value of appliances, farm equipment, vehicles, and land/home ownership. The Test of Non-verbal Intelligence (TONI-IV) was completed by participants as a language free assessment of intellectual ability (35). The study nurse abstracted the following data from medical records at delivery: sex, birth weight and length, head circumference, delivery type, Apgar at 1 and 5 min, and gestational age. All variables from these documents were evaluated as confounders in final statistical models.

### OP insecticide exposure assessment

Urine samples were obtained up to 6 times during pregnancy at each antenatal care visit. Samples were collected in 100-ml polypropylene urine collection containers and were aliquoted and stored in a −20°C freezer until analysis. To characterize exposure across pregnancy while keeping the analytic burden low, urine samples were composited using equal volumes to create early- (0–14 weeks gestation), mid- (>14 weeks through 27 weeks gestation) and late- (>27 weeks gestation) pregnancy samples that roughly corresponded to trimester. Details about sample collection, composite scheme, and DAP metabolite descriptive statistics including intraclass correlation coefficients and spearman correlations of trimester samples can be found in Baumert et al. (5).

Composited urine samples were analyzed for 6 DAP metabolites using a previously validated method which was also cross-validated by gas chromatography-mass spectrometry (36). All samples were randomized using a Fisher-Yates shuffling algorithm prior to analysis to reduce potential batch effects (37, 38). Briefly, 5 mL of urine was spiked with dibutylphosphate (DBP) as a surrogate internal standard, then acidified with hydrochloric acid. The acidified urine was extracted with ethyl acetate: acetone then the DAP metabolites were derivatized to their pentafluorobenzyl phosphate esters. The target DAP derivatives were isolated from the reaction mixture using a hexane extraction. The concentrated extract was analyzed by gas chromatography-flame photoionization detection. Data were quantified using a continuing calibration curve normalizing on the DBP internal standard area. Quality control (QC) samples were derived from 2 separated urine pools: unspiked pooled urine (low concentration QC pool) and spiked pooled urine where DAP metabolites were spiked into the pool at various concentrations (high concentration QC pool). Two blank samples, four QC samples and calibrants were analyzed concurrently with unknown samples in each analytical run. The limits of detection (LOD) were 5 ng/mL (dimethylphosphate; DMP), 1 ng/mL (dimethylthiophosphate; DMTP), 0.5 ng/mL (dimethyldithiophosphate; DMDTP), 1 ng/mL (DEP), 0.125 ng/mL (diethylthiophosphate; DETP), and 0.25 ng/mL (diethyldithiophosphate; DEDTP) and the relative recoveries ranged from 94 to 119%. Relative standard deviations of the QC pools ranged from 4.5 to 12.6% (36). The laboratory also participated in the proficiency testing program administered by the German External Quality Assessment Scheme.

Because of physiologic change during pregnancy, the validity of using creatinine correction for maternal samples has been debated (17, 19, 21, 39). Nevertheless, we chose this approach because it has been used more often in epidemiological studies of pesticide related neurodevelopment. Thus, we can compare our results with other studies directly. In addition and as stated in O’Brien et al. (40), there is no universally accepted measure of urinary dilution. Creatinine was measured by diluting urine samples 1,000-fold with water after spiking with its isotopically labeled analog. Diluted samples were analyzed by liquid chromatography tandem mass spectrometry coupled with electrospray ionization (41). During the analysis of creatinine, a certified reference material (SRM) obtained from the National Institute of Standards and Technology (NIST) was included (NIST SRM 3667).

### NICU network neurobehavioral scale

Five weeks after birth, maternal and infant participants came to SAWASDEE clinics where the research assistant measured infant participants’ weight, length, and head circumference. Maternal participants completed the Depression Anxiety Stress Scale (DASS) (42–44). Early infant neurologic function, behavior, and signs of stress were measured using the NNNS, a tool developed as an assessment for the at-risk infant such as those born prematurely or prenatally exposed to neurotoxic substances. The NNNS measure is appropriate for infants gestational age 30–46 weeks (corrected for conceptional age (45). The NNNS has strong psychometric qualities with good internal and concurrent validity (46). Study nurses, blind to infant exposure and certified by a Brown University certified trainer, administered the NNNS while the mother observed the exam. During the assessment period, nurses met monthly for reliability checks.

We administered the NNNS in a quiet, temperature-controlled room. The test began with a baseline observation of respiration, color, and tone. If the infant was asleep, a sequence of habituation items was presented to measure the infant’s ability to process visual, auditory, and tactile stimuli, and to protect sleep. The habituation package is often omitted due to the sleep requirement. Examination of primitive reflexes, as well as passive and active tone ensues, followed by social interaction components and an assessment of attention. Additional neurological items were completed, followed by a post-exam observation of respiration, color, and tone to end the assessment. Summarization of NNNS raw data results in scores on 13 dimensions: habituation, attention, arousal, self-regulation, special handling needed from the examiner to assist the infant through the exam, quality of movement, excitability, lethargy, non-optimal reflexes, asymmetrical reflexes, hypertonicity, hypotonicity, and stress/abstinence (45).

### Statistical analysis

Frequencies and percentages or means, standard deviations and range are used to describe the distributions of categorical or continuous covariates, respectively, overall and stratified by location. DAP concentrations were converted into summed DAP concentrations, consisting of ∑DEAP, ∑DMAP, and ∑DAP applying the following formulas: ∑DEA*P* = (DEP/154) + (DETP/170) + (DEDTP/186); ∑DMA*P* = (DMP/126) + (DMTP/142) + (DMDTP/158); ∑DA*P* = ∑DMAP + ∑DEAP. Summed DAP concentrations were created from the composited urine samples to represent early, middle, and late pregnancy period for each subject. The average summed DAP concentration was calculated by combining the summed DAP concentrations obtained from each composited urine sample and dividing by the number of composited urine samples (*n* = 3). The mean ∑DAP variable, reported in μmol/L unit, was included in the model to represent average OP exposure across pregnancy for each individual subject (5). These summed DAP concentrations were treated as continuous variables in statistical models.

The distributions of NNNS outcomes are summarized and expressed as means, standard deviations, and percentiles. Because we administered the habituation package to <50% of our sample (*N* = 125) due to the sleep requirement, scores for habituation were omitted from further analysis. We used regression models to determine the association between NNNS outcomes and summed DAP concentrations. In these models, the heavily right-skewed summed DAP concentrations were log-transformed to reduce outsized influence of the higher values on estimated regression parameters. NNNS measures that were continuous (habituation, attention, handling, self-regulation, arousal, lethargy, quality of movement, and stress abstinence) were evaluated using standard linear regression, while counts (excitability, non-optimal reflexes, and asymmetric reflexes) were evaluated with Poisson regression and binary measures (hyper- and hypotonicity) with logistic regression. As such, standardized regression coefficients (equivalent to partial correlations) estimated the effect of summed DAP concentrations on continuous NNNS measures, risk ratios the effects on count measures, and odds ratios the effects on binary measures. Effects were estimated adjusting for creatinine concentrations and then adding other potential confounders. Potential confounders were identified from the demographic information available (see Table 1) as those that were associated with at least one of the NNNS measures and summed DAP concentrations at the 0.10 significance level.

In cases of significant associations, the means, standard deviations, and percentiles of NNNS measures were calculated for low (0–25th percentile), medium (25–75th percentile), and high (>75th percentile) exposure.

Latent Profile Analysis (LPA) using Mplus 7 statistical package identified groups of infants with similar response patterns. This parametric analysis accounted for the distributions of each of the NNNS measures, specifically, normal for continuous, Poisson for count, and logistic for binary (47). The validity of the resulting groupings was confirmed with non-parametric cluster analysis performed on the standardized scores iteratively using Euclidian distances to identify clusters. Logistic regression evaluated the association of pesticide concentrations on profile membership, adjusting for potential confounders.

All analyses except LPA were performed using SAS software, Version 9.4 of the SAS System for Windows (© 2016, SAS Institute Inc).

## Results

Table 2 is a summary of the study participant demographics and values for summed DAP concentrations for the entire study sample and separately for each location (Chom Thong; Fang) from which participants were recruited. As can be seen in Table 2, participants from these locations differed on several demographic and exposure variables. Most of our mothers spoke Thai and were born in Thailand. However, 98% of participants from Chom Thong were born in Thailand while 60% of mothers from Fang district were born in Myanmar. Most of our sample was married and did not report a history of abuse, intimate partner violence, alcohol or recreational drug use during pregnancy. Mother and father education and family income were lower among Fang compared to Chom Thong participants. Most infants had vaginal birth with similar gestational age, weight, length, head circumference and Apgar scores across sites. The highest summed DAP concentrations were observed for participants from Fang.

### OP Metabolites as predictors of NNNS Summary scores.

Table 3 displays descriptive statistics for the NNNS summary scores for all participants followed by Table 4 showing the associations between OP metabolites and NNNS summary scores. The following variables associated with at least one of the 12 NNNS scores and summed DAP concentrations at *p* ≤ 0.10 were included as confounders in models to predict outcomes: father’s education, infant sex, amenities/appliances in home, having at least one previous preterm birth, gestational age, and maternal TONI-IV. Creatinine was included in models as an independent variable. Initially, we evaluated the effects of ∑DAP concentrations on NNNS summary scales for each trimester. We observed lower arousal among infants with higher ∑DAP concentrations during each trimester and less excitability for the second and third trimester exposures for higher ∑DAP concentrations (data not shown). Because we did not find a consistent effect of trimester specific ∑DAP concentrations, the remainder of our analyses used average ∑DAP concentrations across pregnancy.

We observed a statistically significant inverse association between NNNS arousal and log values of both ∑DAP [*β* = −0.10 (CI: −0.17, −0.02) *p* = 0.0091] and ∑DEAP but not ∑DMAP concentrations. Participants had a significantly lower risk of excitability with higher prenatal ∑DAP concentrations [0.79** (0.68, 0.92) *p* = 0.0026] and higher ∑DMAP and ∑DEAP concentrations (see Table 4). To explore sex differences for the associations of OP insecticides on NNNS outcomes, we performed the same regression analyses stratified by infant sex and controlling for covariates and creatinine correction. We observed that lower excitability and arousal with higher ∑DAP concentrations were not different for males and females (data not shown).

To explore further the associations of ∑DAP concentrations on infant arousal and excitability, we grouped participants based on high, medium and low prenatal ∑DAP concentrations (Table 5). Relative to a US normative sample of infants examined at 4 weeks old, our participants’ average NNNS summary scores were within the normative range, defined as between the 10 and 90th percentile. However, mean arousal and excitability scores were below the normative 10th percentile for participants who had high ∑DAP concentrations (48). This low arousal and excitability could be indicative of non-optimal development at 1 month. Neither arousal nor excitability changed significantly from birth to 1 month in a US normative sample (48), suggesting that lower arousal and excitability may remain stable. Moreover, our scores for these scales were also below the 10th percentile observed by Fink (49) in a larger US normative sample evaluated at birth.

**FIGURE 1:**
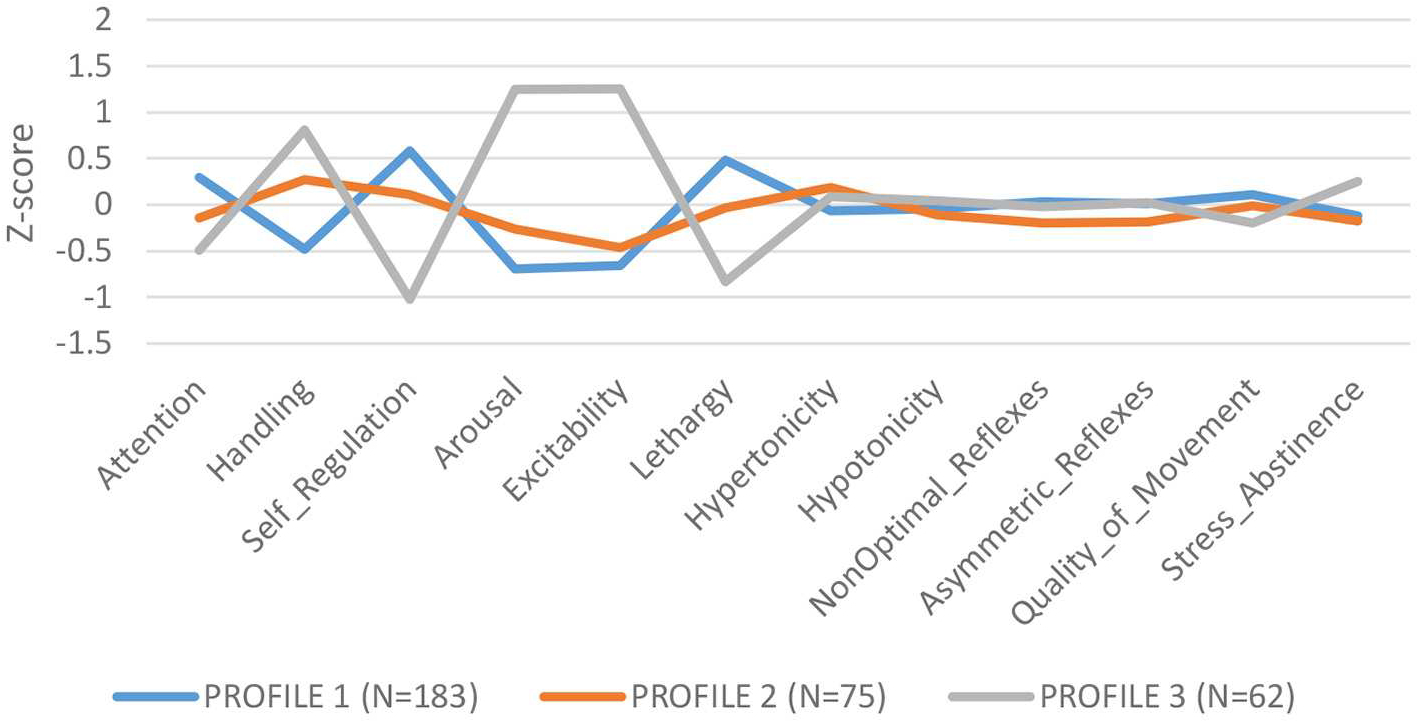
NNNS profiles.

LPA of NNNS summary scales identified 3 profiles: Profile 1 (*N* = 183): low need for handling, self regulated, better attention, and low arousal/excitability; Profile 2 (*N* = 75): need for handling, and moderate levels of arousal, self regulation and excitability; Profile 3 (*N* = 62): higher need for handling, low attention and self-regulation, high arousal and excitability and greater stress/abstinence (see Figure 1). We compared summary scores across profiles and found significant differences for each summary scale, validating the independence of the profiles (Supplementary Table S1).

Overall higher ∑DAP concentrations conferred lower odds of being classified in Profile 3, characterized as infants with lower attention and self-regulation and higher arousal and excitability [Profile 1: OR = 0.55 (CI: 0.34–0.86) *p* = 0.012]. Moreover, overall higher ∑DAP concentrations increased the odds ratio for Profile 1, identified as more self regulated, better attention, but lower excitability and arousal [Profile 1: OR = 1.47 (CI: 1.05–2.06) *p* = 0.03]. ∑DEAP metabolites reflecting chlorpyrifos exposure were responsible for the associations with NNNS profiles while ∑DMAP metabolites were not associated with specific profiles. Higher ∑DAP metabolites in the first and second trimester predicted lower odds of being in Profile 3, reflecting higher excitability and arousal, and greater odds of being categorized in Profile 1, reflecting higher self-regulation and lower excitability (see Supplementary Tables S2a–c). ∑DAP concentrations measured in the third trimester did not impact the odds ratio for any profile.

## Discussion

Although one aim of our study was to determine trimester specific susceptibility, we did not find consistent associations with NNNS scales and ∑DAPs for each trimester. We observed, however, an inverse association between prenatal urinary DAP concentrations averaged across pregnancy and NNNS summary scales for arousal and excitability among 5 week old Thai infants. These infants exhibited lower levels of fussing, crying and associated motor activity throughout the examination (i.e., arousal) and lower levels of motor, state, and physiological reactivity and irritability (i.e., excitability) (50). DEAP metabolites reflecting exposure to pesticides such as chlorpyrifos but not dimethylalkylphosphate metabolites (e.g., malathion) were the OP insecticides predictive of low arousal while both DEAP and DMP metabolites were associated with low excitability. For infants whose maternal metabolite concentrations were >the 75th percentile, over 50 % of those infants scored zero on excitability and 50% scored below the normative value for arousal (48, 49). Arousal and excitability scores that are extremely high or low may confer risk for achieving neurodevelopmental milestones. In contrast, profile analysis did not reveal a significant association between higher prenatal ∑DAP concentrations and Profile 3, a higher risk profile reflecting lower attention, self-regulation and higher arousal and excitability. Instead, infants with higher prenatal OP insecticide exposure, particularly during the first and second trimester were more likely to be within Profile 1, reflecting better attention and self-regulation but lower arousal and excitability. These disparate results imply that higher OP insecticide exposure for some infants may be protective based on better attention and self-regulation. Future analyses of neurodevelopmental measures of visual attention, processing speed, and memory administered at 4 and 7 months will test the neurodevelopmental implications of low excitability and arousal observed at 5 weeks.

OP insecticides inhibit AChE, the enzyme that breaks down the neurotransmitter, acetylcholine (Ach), and is found in both the peripheral and central nervous systems (51). The acute effects of OP insecticide poisoning are well known, but whether neurodevelopmental effects are observed among infants and children in the absence of AChE inhibition evidence remains controversial. Several previous US birth cohort studies have shown mixed results of prenatal OP insecticide exposure on early infant behaviors. In US birth cohorts of 2–3 day old infants evaluated with the NBAS, higher concentrations of DAPs during pregnancy were associated with more abnormal reflexes (14, 22). Yolton et al. (31), however, observed that 5 week old US infants with higher prenatal exposure to OP insecticides producing DEAP metabolites had improved attention, lower lethargy and less hypotonia while ∑DAP concentrations were associated with fewer signs of autonomic distress. These disparate findings may be due to assessments performed a few days after birth relative to 5 weeks of age when the trauma of birth is resolved. However, it is also noteworthy that ∑DAP concentrations vary significantly among birth cohorts which is also likely to contribute to variability in outcomes. For example, the concentrations observed in our occupational cohort were higher than previous US birth cohorts from California (CHAMACOS: 111.7 nmol/g creatinine), Cincinnati (HOME:76.17 nmol/g creatinine), and New York (Columbia: 39.16 nmol/g creatinine; Mount Sinai:73.8 nmol/g creatinine) (7, 19, 20, 52).

Overall, our NNNS profile analysis reveals that higher ∑DAPs are associated with a profile reflecting better self regulation and attention, and less need for handling but lower arousal and excitability. This outcome may be consistent with Yolton et al. (31) who reported no detrimental effects of OP exposure during gestation and also observed improved attention and reduced lethargy. However, our observation of a lower level of arousal and excitability while potentially reflecting stability in behavior, very low arousal and excitability can also suggest that infants may not respond adequately to stimulation. Our findings show similarity with other studies of infants exposed prenatally to drugs of abuse and implicate effects on the dopaminergic and serotonergic systems. For example, infants exposed prenatally to methamphetamine (MA) exhibited lower arousal and excitability along with poorer quality of movement, and more total stress/abstinence (53). Prenatal cocaine exposure also resulted in lower arousal and inability to self-regulate among 1 month old infants evaluated with the NNNS (54). A study of African American infants with prenatal exposures to the stimulant nicotine (higher cotinine) exhibited decreased arousal and excitability along with increased self-regulation and hypotonicity in contrast to white infants who exhibited the opposite effect (55). African American infants were less responsive during the examination suggesting reduced neurobehavioral activation (55).

Rodent studies of MA exposure suggest toxic effects on dopaminergic and serotonergic neurons, both systems also shown to be sensitive to OP insecticide exposure in animal models (8–10). However, findings for drugs of abuse are not universal. For example, prenatal, self-report exposure to cocaine, alcohol, tobacco, and marijuana were predictive of increased arousal and excitability, hypertonia, poor quality of movement, self-regulation and attention (56). The latter study, however, was based largely on self-report rather than biomarkers of exposures.

Scores for excitability and arousal among OP insecticide exposed infants in the present study were lower than infants with prenatal exposure to drugs of abuse and for premature infants reported to have low excitability scores (57). However, comparisons to US normative data for the NNNS may not be applicable for Thai infants. Nevertheless, we have a relatively large sample size and have taken into account a full suite of potential confounders to evaluate the effects of prenatal OP insecticide exposures. Future validation of our NNNS results for neurodevelopmental outcomes will await analysis of neurodevelopmental measures, including measures of attention, memory, executive function and overall cognitive and motor development up to 3 years of age.

## Supplementary Material

Supplement

## Figures and Tables

**TABLE 1:** Eligibility criteria for pregnant women and Infants, study of Asian women and their offspring’s’ development and environmental exposure (2017–2019).

Pregnant women eligibility criteria	Infant eligibility criteria

1. 18–40 years old 2. Estimated gestational age (LMP) ≤16 weeks 3. Agricultural worker or live within 50 m of agricultural field 4. Have Thai identification card 5. Reside in district ≥ 6 month, plan delivery at Chom Thong or Fang hospitals and maintain residence > 3 years after delivery 6. Healthy (i.e., no major medical conditions such as hypertension, diabetes, thyroid disease, HIV) 7. consumed fewer than two alcoholic beverages (beer, wine, liquor) per day and did not use illegal drugs 8. Understand and speak Thai 9. Singleton pregnancy	1. Birth, gestation more than 32 weeks 2. Baby weight after birth more than 1,500 g 3.Able to come to NNNS within 45 days after birth 4. Healthy (no anomaly)

**TABLE 2:** Overall and location specific family and infant demographics and ΣDAP values, study of Asian women and their offsprings’ development and environmentalexposure (2017–2019)*.

Variable	Levels	Overall *N* = 320)** *n*(%)	Chom thong (*N* = 215) *n* (%)	Fang (*N* = 105) *n* (%)

Maternal birth country	Thailand	272 (86%)	210(98%)	41 (40%)
	Myanmar	45 (14%)	4 (2%)	62 (60%)
Maternal Thai language ability	Non-Thai (translator)	5 (2%)	1 (<1%)	4 (4%)
	Understand; can’t	70 (22%)	12 (6%)	58 (56%)
	read/write			
	Understand, read/write	243 (76%)	201 (94%)	42 (40%)
Marital status	Legally Married	47(14%)	46 (21%)	1 (1%)
	Living as Married	267 (83%)	165 (77%)	102 (97%)
	Separated	2 (1%)	0 (0%)	2 (2%)
	Widowed	2 (1%)	2 (1%)	0 (0%)
	Divorced	2 (1%)	2 (1%)	0 (0%)
Maternal education	None	49(16%)	10 (5%)	39 (42%)
	Some elementary	17 (6%)	6 (3%)	11 (12%)
	Elementary	35 (12%)	20 (9%)	15 (16%)
	Lower Intermediate	95 (32%)	78 (38%)	17(19%)
	Upper intermediate or college	103 (34%)	93 (45%)	10(11%)
Paternal education	None	40(14%)	6 (3%)	34 (43%)
	Some elementary	13 (5%)	6 (3%)	7 (9%)
	Elementary	58 (20%)	36(17%)	22 (27%)
	Lower Intermediate	91 (32%)	83 (40%)	8(10%)
	Upper intermediate or college	85 (29%)	76 (37%)	9 (11%)
Prior preterm birth	Yes	20 (6%)	13 (6%)	7 (7%)
	No	295 (94%)	197 (94%)	98 (93%)
Delivery type	Vaginal	256 (81%)	170 (80%)	86 (82%)
	Cesarean section	62 (19%)	43 (20%)	19(18%)
Infant sex	Male	158 (49%)	105 (49%)	53 (50%)
	Female	162 (51%)	110(51%)	52 (50%)
		Mean (SD) Min – Max	Mean (SD) Min – Max	Mean (SD) Min – Max
Maternal age	*N* = 320	25.1 (5.3) 18–39	25.6(5.3) 18–39	24.0 (5.1) 18–38
Maternal years education	*N* = 299	8.1 (4.6) 0–16	9.8 (3.5) 0–16	4.4 (4.5) 0–16
Paternal years education	*N* = 287	8.0 (4.3) 0–16	9.5 (3.2) 0–16	4.3 (4.4) 0–16
TONI4_Index (IQ) Maternal	*N* = 294	82.1 (8.9) 60–109	84.3 (8.1)	77.0 (8.6)
			60–109	61–96
Income/month (Thai Baht)	*N* = 284	10,641 (9,661) 500–60,000	11,944 (10,525) 800–60,000	7,262 (5,735) 500–30,000
ΣDAP (nmol/L) Early pregnancy	*N* = 289	160.93 (319.00)	100.28 (102.15)	277.34 (508.04)
		41.39–4139.00	41.39–1104.00	41.39–4139.00
ΣDAP (nmol/L) Mid-pregnancy	*N* = 305	162.27 (348.90)	93.45 (69.18) 41.39–455.23	305.47 (580.45)
		41.39–4,856.00		44.75–4856.00
ΣDAP (nmol/L) Late pregnancy	*N* = 305	146.67 (479.76)	129.40 (572.12)	182.61 (165.45)
		41.39–8,221.00	41.39–8221.00	44.28–866.95
ΣDAP (nmol/L) averaged over pregnancy	*N* = 319	154.8 (244.9) 42.6–2,852.1	108.1 (197.4) 42.6–2852.1	251.2 (300.5) 43.9–1942.3
ΣDEAP (nmol/L) averaged over pregnancy	*N* = 319	99.9 (181.8) 7.3–1,714.4	53.0 (51.5) 7.3–511.3	196.8 (287.2) 8.6–1714.4
ΣDMAP (nmol/L) averaged over pregnancy	*N* = 319	54.9 (155.6) 35.3–2,758.4	55.2 (186.9) 35.3–2758.4	54.3 (46.8) 35.3–275.4
Maternal pregnancy weight gain (kg)	*N* = 289	9.9 (5.0) −4 – +35	10.5 (4.9)−3 – +35	8.9 (5.0) −4 – +23.0
Gestational age (weeks)	*N* = 319	38.5 (1.1) 34.6–41.0	38.5 (1.1) 34.6–41.0	38.5 (1.1) 34.6–40.3
Infant weight (kg)	*N* = 319	3.00 (0.41) 1.73–4.22	3.03 (0.43) 1.73–4.22	2.94 (0.37) 1.95–3.97
Infant length (cm)	*N* = 316	48.4 (2.6) 37–55	47.6 (2.4) 37–55	50.0 (2.2) 44–55
Infant head circumference (cm)	*N* = 313	32.8 (1.5) 29–39	32.6 (1.5) 29–39	33.3 (1.6) 30–37
Apgar score at 1 minute	*N* = 313	8.9 (0.8) 4–10	8.9 (0.8) 5–10	8.8 (0.7) 5–10
Apgar score at 5 minutes	*N* = 313	9.9 (0.4) 7–10	9.8 (0.5) 7–10	>9.9 (0.2) 9–10

* The following variables with low values are excluded from the Table: maternal abuse and intimate partner violence, maternal alcohol, smoking, & drug use; maternal depression, stress, anxiety.

** Not all cell counts within a variable sum to total N in column header because of missing values. For continuous variables (for which mean, standard deviation, and range are given), the number of observed values are included in the second column.

**TABLE 3:** Descriptive statistics for NNNS summary scores, study of Asian women and their offsprings’ development and environmental exposure (2017–2019).

NNNS scores	*M*	SD	Min	Max	10th	25th	50th	75th	90th

Attention	5.71	0.79	1.71	7.29	4.86	5.29	5.71	6.14	6.57
Arousal	3.67	0.54	2.71	5.29	3.00	3.29	3.57	4.00	4.29
Regulation	6.08	0.58	4.58	7.36	5.27	5.67	6.13	6.54	6.83
Handling	0.18	0.24	0	0	0	0.13	0.13	0.25	0.50
Quality of movements	5.51	0.23	4.60	6.20	5.2	5.50	5.50	5.67	5.67
Excitability	1.20	1.59	0	7.00	0	0	0	2.00	4.00
Lethargy	3.19	1.11	1.00	8.00	2	2.00	3.00	4.00	4.00
Nonoptimal reflexes	4.13	1.33	0	8.00	3	3.00	4.00	5.00	6.00
Asymmetrical Reflexes	0.46	0.71	0	4.00	0	0	0	1.00	1.00
Stress/abstinence	0.06	0.03	0	0.16	0.02	0.04	0.06	0.08	0.10
	Yes	No							
	*N*(%)	*N*(%)							
Hyper	11 (3.4%)	309 (96.6%)							
Hypo	23 (7.2%)	297 (92.8%)							

**TABLE 4:** Associations of averaged DAP concentrations and NNNS summary scores*.

NNNS	Log ΣDAP		Log ΣDEAP		Log ΣDMAP	
	Unadjusted	Adjusted	Unadjusted	Adjusted	Unadjusted	Adjusted

**Linear models**						
Regression coefficient (95% CI)						
partial correlation, and *p*-value						
Attention	0.03 (−0.06, 0.12)	0.03 (−0.08, 0.14)	0.02 (−0.08, 0.12)	0.02 (−0.10, 0.13)	0.01 (−0.02, 0.05)	0.01 (−0.02, 0.05)
	0.035, 0.54	0.081, 0.56	0.022, 0.70	0.077, 0.78	0.051, 0.37	0.050, 0.39
Handling	0.02 (0.0, 0.05)	0.03 (−0.01, 0.06)	0.02 (−0.01, 0.06)	0.02 (−0.01, 0.06)	0.0 (−0.01, 0.01)	0.0 (−0.01, 0.01)
	0.093, 0.10	0.085, 0.14	0.084, 0.14	0.071, 0.22	0.018, 0.76	0.021, 0.71
Self-regulation	0.08 (0.00, 0.15)	0.05 (−0.03, 0.13)	0.07 (−0.01, 0.15)	0.05 (−0.04, 0.13)	0.02 (−0.003, 0.046)	0.01 (−0.01, 0.04)
	0.12, 0.039	0.064, 0.26	0.100, 0.074	0.058, 0.31	0.095, 0.091	0.063, 0.27
Arousal	−0.14 (−0.21, −0.07)	−0.10 (−0.17, −0.02)	−0.17 (−0.23, −0.09)	−0.13 (−0.21, −0.05)	−0.01 (−0.04, 0.01)	−0.01 (−0.03, 0.02)
	−0.231, <0.0001	−0.148, 0.0091	−0.248, <0.0001	−0.174, 0.0020	−0.066, 0.24	−0.025, 0.66
Lethargy	0.05 (−0.08, 0.19)	−0.05 (−0.20, 0.10)	0.10 (−0.05, 0.25)	0.01 (−0.16, 0.17)	−0.01 (−0.06, 0.04)	−0.02 (−0.07, 0.03)
	0.042, 0.46	−0.034, 0.55	0.071, 0.20	0.005, 0.93	−0.019, 0.73	−0.047, 0.40
Quality of movement	−0.01 (−0.04, 0.02)	−0.03 (−0.06, 0.01)	−0.02 (−0.05, 0.01)	−0.03 (−0.07, 0.002)	0.00 (−0.01, 0.01)	0.00 (−0.01, 0.01)
	−0.040, 0.47	−0.003, 0.12	−0.059, 0.29	−0.104, 0.068	0.007, 0.90	0.026, 0.82
Stress abstinence	−0.01 (−0.01,0.002)	−0.002 (−0.007, 0.002)	−0.01, (−0.01, −0.003)	0.00 (−0.01, 0.001)	0.0 (−0.001, 0.001)	0.0 (−0.001, 0.002)
	−0.172, 0.0021	−0.119, 0.28	−0.189, 0.0007	0.080, 0.16	0.018, 0.76	0.017, 0.76
**Poisson models**						
Rate ratio, 95% confidence interval, *p*-value						
Excitability	0.75 (0.72, 0.79)	0.79** (0.68, 0.92)	0.76 (0.73, 0.79)	0.80 (0.68, 0.93)	0.93 (0.91, 0.95)	0.94 (0.89, 1.00)
	<0.0001	0.0026	<0.0001	0.0035	<0.0001	0.049
Non-optimal reflexes	1.04 (1.02, 1.06)	1.03 (0.96, 1.10)	1.04 (1.02, 1.06)	1.03 (0.95, 1.10)	1.01 (1.003, 1.02)	0.01 (0.99, 1.03)
	<0.0001	0.45	0.0003	0.50	0.0042	0.56
Asymmetric reflexes	0.88 (0.82, 0.93)	0.94 (0.76, 1.17)	0.78 (0.72, 0.83)	0.82 (0.65, 1.05)	1.01 (0.99, 1.03)	1.02 (0.96, 1.08)
	<0.0001	0.59	<0.0001	0.11	0.16	0.49
**Logistic models**						
OR (95% CI)						
*p*-value						
Hypertonicity	0.78 (0.43, 1.39)	0.92 (0.47, 1.80)	0.77 (0.39, 1.51)	0.86 (0.39, 1.94)	0.89 (0.78, 1.02)	0.93 (0.81, 1.08)
	0.40	0.80	0.44	0.72	0.10	0.37
Hypotonicity	1.18 (0.69, 2.15)	1.08 (0.60, 1.97)	1.25 (0.70, 2.24)	1.15 (0.59, 2.23)	1.01 (0.85, 1.21)	1.00 (0.84, 1.19)
	0.53	0.80	0.46	0.67	0.88	0.97

* Covariates used in all models: father’s education, infant sex, amenities/appliances in home, having at least one previous preterm birth, gestational age, and maternal TONI-IV; creatinine entered in models as an independent variable.

** Number of excitability characteristics among those at the 75th percentile of exposure is 21% less than the number of characteristics at the 25th percentile.

**TABLE 5:** NNNS arousal and excitability summary scores in high, medium, and low ΣDAP concentration categories.

ΣDAP	Mean (SD)	Minimum	10 th	25th	50th	75th	90th	Maximum

**Descriptive statistics for arousal summary score**								
Low = <25% (*N* = 80) (42.6–<64.3)	3.79 (0.59)	3.00	3.00	3.29	3.71	4.29	4.71	5.29
Medium 25–75% (*N* = 161) (64.3–<141.9)	3.72 (0.54)	2.71	3.00	3.29	3.57	4.14	4.57	5.14
High >75% (*N* = 79) (141.9–2852.1)	3.44 (0.38)	2.86	3.00	3.14	3.43	3.71	4.00	4.43
**Descriptive statistics for excitability summary score**								
Low (*N* = 80) (<25%, 42.6–<64.3)	1.53 (0.60)	0	0	0	1	3	4	7
Medium (*N* = 161) (25–75%, 64.3–<141.9)	1.23 (1.62)	0	0	0	0	2	4	5
High (*N* = 79) (>75%, 141.9–2852.1)	0.81 (1.22)	0	0	0	0	1	2	5

## Data Availability

The raw data supporting the conclusions of this article will be made available by the authors, without undue reservation.

## SAWASDEE birth cohort investigative team

Dana Boyd Barr, Nancy Fiedler, Tippawan Prapamontol, Panrapee Suttiwan, Warangkana Naksen, Parinya Panuwet, P. Barry Ryan, Kyle Steenland, Melissa M. Smarr, Brittney O. Baumert, Priya D’Souza, Grace E. Lee, Volha Yakimavets, Tamaria Hawkins, Margaret Sullivan, Pamela Ohman-Strickland, Mark G. Robson, Sompoch Iamsupasit, Wattasit Siriwong, Kathryn J. Barr, Rachel Greenwald, Carol Cheatham, Ampica Mangklabruks, Tanyaporn Kerdnoi, Namtip Srirak, Surat Hongsibsong, Supattra Sittiwang, Pimjuta Nimmapirat, Wathoosiri Promduang, Natabhol Jayakittivaraloes, Nattawadee Promkam, Sureewan Pingwong, Kewalin Kunsupa, Anchana Kantasri, Kritsanachai Kaewthit, Anchalee Wongkampaun, Nathaporn Thongjan, Kanyapak Kohsuwan, Ranuka Dawandee, Maytinee Chaimidchid, Sasiwimon Soonsawat, Kingpaka Sritongkom, Sakawrat Namakunna, and Soythong Pinasu.
