# Supplementary material for: The relationship between prenatal exposure to organophosphate insecticides and neurodevelopmental integrity of infants at 5-weeks of age
Source: Front Epidemiol. 2022 Dec 14;2:1039922. doi: 10.3389/fepid.2022.1039922 (PMC10016628; doi:10.3389/fepid.2022.1039922)
Supplement: Supplementary file 1 [file Table_1.docx]

Supplementary Materials

**Table S1:** Analysis of variance to compare NNNS profiles, Study of Asian Women and their Offsprings’ Development and Environmental Exposure (2017-2019)

|  | Profile 1 | Profile 2 | Profile 3 | p value |
| --- | --- | --- | --- | --- |
|  | Mean (SD)  range | Mean(SD)  range | Mean(SD)  range |  |
| Attention | 5.94 (0.71)  2.57-7.29 | 5.37 (0.88)  1.71-7.14 | 5.43 (0.67)  3.00-6.71 | <.0001 |
| Handling | 0.07 (0.14)  0-0.75 | 0.36 (0.27)  0-0.88 | 0.31 (0.23)  0-1.00 | <.0001 |
| Self-Regulation | 6.42 (0.40)  5.14-7.36 | 5.81 (0.43)  4.85-6.87 | 5.43 (0.42)  4.58-6.40 | <.0001 |
| Arousal | 3.30 (0.25)  2.71-4.00 | 3.90 (0.22)  3.43-4.43 | 4.48 (0.34)  3.71-5.29 | <.0001 |
| Excitability | 0.15 (0.41)  0-2 | 1.52 (0.78)  0-3 | 3.94 (0.99)  2-7 | <.0001* |
| Lethargy | 3.73 (0.76)  2-8 | 2.61 (1.35)  2-8 | 2.31 (0.64)  1-5 | <.0001 |
| Hypertonicity | 0.06 (0.25)  0-1 | 0.08 (0.27)  0-1 | 0.02 (0.13)  0-1 | 0.070** |
| Hypotonicity | 0.06 (0.25)  0-1 | 0.05 (0.23)  0-1 | 0.11 (0.32)  0-1 | 0.37** |
| NonOptimal_Reflexes | 4.36 (1.20)  1-7 | 4.00 (1.56)  0-8 | 4.54 (1.34)  2-7 | 0.039* |
| Asymmetric_Reflexes | 0.48 (0.69)  0-3 | 0.33 (0.55)  0-2 | 0.63 (0.91)  0-4 | 0.050* |
| Quality_of_Movement | 5.54 (0.22)  4.60-5.83 | 5.48 (0.22)  4.67-5.83 | 5.47 (0.28)  4.67-6.20 | 0.065 |
| Stress_Abstinence | 0.052 (0.029)  0-0.14 | 0.053 (0.034)  0-0.14 | 0.069 (0.032)  0-0.16 | 0.0007 |

* Calculated via Poisson regression for count variables*

**Calculated via logistic regression for binary variables

| **Table S2a:** Covariate and creatinine adjusted^*^ odds Ratios for PROFILE 1: High self-regulation/low excitability and arousal per inter-quartile increase in pesticide biomarkers, Study of Asian Women and their Offsprings’ Development and Environmental Exposure (2017-2019) | | | | | | | | | | | | | | | | | | | |
| --- | --- | --- | --- | --- | --- | --- | --- | --- | --- | --- | --- | --- | --- | --- | --- | --- | --- | --- | --- |
|  |  | | | | **Odds Ratio** | | **95% Confidence Limits** | | | | | | **p-value** | | | |  | | |
| OVERALL | **Log** ∑**DAP** | | | | 1.47 | | 1.05- 2.06 | | | | 0.026 | | | |  | | | |  |
|  | **Log** ∑**DEAP** | | | | 1.73 | | 1.05-2.85 | | | | 0.032 | | | |  | | | |  |
|  | **Log** ∑**DMAP** | | | | 1.02 | | 0.99-1.05 | | | | 0.23 | | | |  | | | |  |
| TRIMESTER 1 | **Log** ∑**DAP 1** | | | | 1.57 | | 1.02-2.42 | | | | 0.041 | | | |  | | | |  |
|  | **Log** ∑**DEAP 1** | | | | 1.51 | | 0.97-2.34 | | | | 0.070 | | | |  | | | |  |
|  | **Log** ∑**DMAP 1** | | | | 1.29 | | 0.92-1.82 | | | | 0.14 | | | |  | | | |  |
| TRIMESER 2 | **Log** ∑**DAP 2** | | | | 1.69 | | 1.05-2.73 | | | | 0.030 | | | |  | | | |  |
|  | **Log** ∑**DEAP 2** | | | | 1.83 | | 1.14-2.93 | | | | 0.012 | | | |  | | | |  |
|  | **Log** ∑**DMAP 2** | | | | 1.33 | | 0.72-2.46 | | | | 0.36 | | | |  | | | |  |
| TRIMESTER 3 | **Log** ∑**DAP 3** | | | | 1.21 | | 0.83-1.78 | | | | 0.33 | | | |  | | | |  |
|  | **Log**∑ **DEAP 3** | | | | 1.33 | | 0.89-2.01 | | | | 0.17 | | | |  | | | |  |
|  | **Log** ∑**DMAP 3** | | | | 0.98 | | 0.72-1.33 | | | | 0.87 | | | |  | | | |  |
| **Table S2b:** Covariate and creatinine adjusted^*^ odds ratios for PROFILE 2: Moderate arousal and need for handling per inter-quartile increase in pesticide biomarkers, Study of Asian Women and their Offsprings’ Development and Environmental Exposure (2017-2019) | | | | | | | | | | | | | | | | | | |  |
|  | | |  | | | | **Odds Ratio** | | **95% Confidence Limits** | | | | | | **p value** | |  | |  |
| OVERALL | | | **Log** ∑**DAP** | | | | 0.95 | | 0.65 | | 1.38 | | | | 0.77 | |  | |  |
|  | | | **Log** ∑**DEAP** | | | | 0.96 | | 0.54 | | 1.70 | | | | 0.88 | |  | |  |
|  | | | **Log** ∑**DMAP** | | | | 0.99 | | 0.96 | | 1.02 | | | | 0.46 | |  | |  |
| TRIMESTER 1 | | | **Log** ∑**DAP 1** | | | | 0.98 | | 0.61 | | 1.58 | | | | 0.93 | |  | |  |
|  | | | **Log** ∑**DEAP 1** | | | | 1.03 | | 0.62 | | 1.69 | | | | 0.92 | |  | |  |
|  | | | **Log** ∑**DMAP 1** | | | | 0.88 | | 0.60 | | 1.29 | | | | 0.52 | |  | |  |
| TRIMESTER 2 | | | **Log** ∑**DAP 2** | | | | 0.95 | | 0.56 | | 1.59 | | | | 0.84 | |  | |  |
|  | | | **Log** ∑**DEAP 2** | | | | 0.92 | | 0.54 | | 1.56 | | | | 0.76 | |  | |  |
|  | | | **Log** ∑**DMAP 2** | | | | 0.80 | | 0.39 | | 1.67 | | | | 0.56 | |  | |  |
| TRIMESTER 3 | | | **Log** ∑**DAP 3** | | | | 1.01 | | 0.65 | | 1.57 | | | | 0.96 | |  | |  |
|  | | | **Log** ∑**DEAP 3** | | | | 0.99 | | 0.62 | | 1.60 | | | | 0.98 | |  | |  |
|  | | | **Log** ∑**DMAP 3** | | | | 1.05 | | 0.72 | | 1.51 | | | | 0.81 | |  | |  |
|  | | |  |  | | | | |  | | |  |  |  |  |  |  |  |  |

| **Table S2c**: Covariate and creatinine adjusted^*^ odds ratios for PROFILE 3: Low attention, self-regulation and high excitability/arousal per inter-quartile increase in pesticide biomarkers, Study of Asian Women and their Offsprings’ Development and Environmental Exposure (2017-2019) | | | | | | |  | |  |
| --- | --- | --- | --- | --- | --- | --- | --- | --- | --- |
|  |  |  | **Odds Ratio** | **95% Confidence Limits** | | **p value** | |  |  |
| OVERALL | **Log** ∑**DAP** |  | 0.55 | 0.34 | 0.88 | 0.012 | |  |  |
|  | **Log** ∑**DEAP** |  | 0.45 | 0.24 | 0.85 | 0.013 | |  |  |
|  | **Log** ∑**DMAP** |  | 0.99 | 0.95 | 1.02 | 0.41 | |  |  |
| TRIMESTER 1 | **Log** ∑**DAP 1** |  | 0.45 | 0.24 | 0.85 | 0.013 | |  |  |
|  | **Log** ∑**DEAP 1** |  | 0.50 | 0.28 | 0.89 | 0.019 | |  |  |
|  | **Log** ∑ **DMP 1** |  | 0.73 | 0.43 | 1.22 | 0.22 | |  |  |
| TRIMESTER 2 | **Log** ∑**DAP 2** |  | 0.43 | 0.22 | 0.84 | 0.013 | |  |  |
|  | **Log** ∑**DEAP 2** |  | 0.44 | 0.24 | 0.79 | 0.0065 | |  |  |
|  | **Log** ∑**DMAP 2** |  | 0.80 | 0.37 | 1.75 | 0.58 | |  |  |
| TRIMESTER 3 | **Log** ∑**DAP 3** |  | 0.72 | 0.44 | 1.19 | 0.20 | |  |  |
|  | **Log** ∑**DEAP 3** |  | 0.65 | 0.39 | 1.09 | 0.10 | |  |  |
|  | **Log** ∑**DMAP 3** |  | 0.99 | 0.66 | 1.47 | 0.94 | |  |  |

^*^Covariates used in all models: father’s education, infant sex, amenities/appliances in home, having at least one previous preterm birth, gestational age, and maternal TONI-IV; creatinine included in models as an independent variable.
