# Supplementary material for: The relationship between prenatal exposure to organophosphate insecticides and neurodevelopmental integrity of infants at 5-weeks of age
Source: Front Epidemiol. Author manuscript; Available in PMC 2023 Mar 15. (PMC10016628; doi:10.3389/fepid.2022.1039922)
Supplement: Supplementary file 1 [file NIHMS1872363-supplement-Supplement.docx]

Supplementary Materials

**Table S1:** Analysis of variance to compare NNNS profiles, Study of Asian Women and their Offsprings’ Development and Environmental Exposure (2017-2019)

|  | Profile 1 | Profile 2 | Profile 3 | p value |
| --- | --- | --- | --- | --- |
|  | Mean (SD) range | Mean(SD) range | Mean(SD) range |  |
| Attention | 5.94 (0.71) 2.57-7.29 | 5.37 (0.88) 1.71-7.14 | 5.43 (0.67) 3.00-6.71 | <.0001 |
| Handling | 0.07 (0.14) 0-0.75 | 0.36 (0.27) 0-0.88 | 0.31 (0.23) 0-1.00 | <.0001 |
| Self-Regulation | 6.42 (0.40) 5.14-7.36 | 5.81 (0.43) 4.85-6.87 | 5.43 (0.42) 4.58-6.40 | <.0001 |
| Arousal | 3.30 (0.25) 2.71-4.00 | 3.90 (0.22) 3.43-4.43 | 4.48 (0.34) 3.71-5.29 | <.0001 |
| Excitability | 0.15 (0.41) 0-2 | 1.52 (0.78) 0-3 | 3.94 (0.99) 2-7 | <.0001* |
| Lethargy | 3.73 (0.76) 2-8 | 2.61 (1.35) 2-8 | 2.31 (0.64) 1-5 | <.0001 |
| Hypertonicity | 0.06 (0.25) 0-1 | 0.08 (0.27) 0-1 | 0.02 (0.13) 0-1 | 0.070** |
| Hypotonicity | 0.06 (0.25) 0-1 | 0.05 (0.23) 0-1 | 0.11 (0.32) 0-1 | 0.37** |
| NonOptimal_Reflexes | 4.36 (1.20) 1-7 | 4.00 (1.56) 0-8 | 4.54 (1.34) 2-7 | 0.039* |
| Asymmetric_Reflexes | 0.48 (0.69) 0-3 | 0.33 (0.55) 0-2 | 0.63 (0.91) 0-4 | 0.050* |
| Quality_of_Movement | 5.54 (0.22) 4.60-5.83 | 5.48 (0.22) 4.67-5.83 | 5.47 (0.28) 4.67-6.20 | 0.065 |
| Stress_Abstinence | 0.052 (0.029) 0-0.14 | 0.053 (0.034) 0-0.14 | 0.069 (0.032) 0-0.16 | 0.0007 |

* Calculated via Poisson regression for count variables* 

**Calculated via logistic regression for binary variables

| **Table S2a:** Covariate and creatinine adjusted^*^ odds Ratios for PROFILE 1: High self-regulation/low excitability and arousal per inter-quartile increase in pesticide biomarkers, Study of Asian Women and their Offsprings’ Development and Environmental Exposure (2017-2019) |  |  |  |  |  |  |  |  |  |  |  |  |  |  |  |  |
| --- | --- | --- | --- | --- | --- | --- | --- | --- | --- | --- | --- | --- | --- | --- | --- | --- |
|  |  |  | **Odds Ratio** | **95% Confidence Limits** |  |  |  |  |  | **p-value** |  |  |  |  |  |  |
| OVERALL | **Log** ∑**DAP** |  | 1.47 | 1.05- 2.06 |  |  |  | 0.026 |  |  |  |  |  |  |  |  |
|  | **Log** ∑**DEAP** |  | 1.73 | 1.05-2.85 |  |  |  | 0.032 |  |  |  |  |  |  |  |  |
|  | **Log** ∑**DMAP** |  | 1.02 | 0.99-1.05 |  |  |  | 0.23 |  |  |  |  |  |  |  |  |
| TRIMESTER 1 | **Log** ∑**DAP 1** |  | 1.57 | 1.02-2.42 |  |  |  | 0.041 |  |  |  |  |  |  |  |  |
|  | **Log** ∑**DEAP 1** |  | 1.51 | 0.97-2.34 |  |  |  | 0.070 |  |  |  |  |  |  |  |  |
|  | **Log** ∑**DMAP 1** |  | 1.29 | 0.92-1.82 |  |  |  | 0.14 |  |  |  |  |  |  |  |  |
| TRIMESER 2 | **Log** ∑**DAP 2** |  | 1.69 | 1.05-2.73 |  |  |  | 0.030 |  |  |  |  |  |  |  |  |
|  | **Log** ∑**DEAP 2** |  | 1.83 | 1.14-2.93 |  |  |  | 0.012 |  |  |  |  |  |  |  |  |
|  | **Log** ∑**DMAP 2** |  | 1.33 | 0.72-2.46 |  |  |  | 0.36 |  |  |  |  |  |  |  |  |
| TRIMESTER 3 | **Log** ∑**DAP 3** |  | 1.21 | 0.83-1.78 |  |  |  | 0.33 |  |  |  |  |  |  |  |  |
|  | **Log**∑ **DEAP 3** |  | 1.33 | 0.89-2.01 |  |  |  | 0.17 |  |  |  |  |  |  |  |  |
|  | **Log** ∑**DMAP 3** |  | 0.98 | 0.72-1.33 |  |  |  | 0.87 |  |  |  |  |  |  |  |  |
| **Table S2b:** Covariate and creatinine adjusted^*^ odds ratios for PROFILE 2: Moderate arousal and need for handling per inter-quartile increase in pesticide biomarkers, Study of Asian Women and their Offsprings’ Development and Environmental Exposure (2017-2019) |  |  |  |  |  |  |  |  |  |  |  |  |  |  |  |  |
|  |  |  | **Odds Ratio** |  | **95% Confidence Limits** |  |  |  |  |  | **p value** |  |  |  |  |  |
| OVERALL | **Log** ∑**DAP** |  | 0.95 |  | 0.65 |  | 1.38 |  |  |  | 0.77 |  |  |  |  |  |
|  | **Log** ∑**DEAP** |  | 0.96 |  | 0.54 |  | 1.70 |  |  |  | 0.88 |  |  |  |  |  |
|  | **Log** ∑**DMAP** |  | 0.99 |  | 0.96 |  | 1.02 |  |  |  | 0.46 |  |  |  |  |  |
| TRIMESTER 1 | **Log** ∑**DAP 1** |  | 0.98 |  | 0.61 |  | 1.58 |  |  |  | 0.93 |  |  |  |  |  |
|  | **Log** ∑**DEAP 1** |  | 1.03 |  | 0.62 |  | 1.69 |  |  |  | 0.92 |  |  |  |  |  |
|  | **Log** ∑**DMAP 1** |  | 0.88 |  | 0.60 |  | 1.29 |  |  |  | 0.52 |  |  |  |  |  |
| TRIMESTER 2 | **Log** ∑**DAP 2** |  | 0.95 |  | 0.56 |  | 1.59 |  |  |  | 0.84 |  |  |  |  |  |
|  | **Log** ∑**DEAP 2** |  | 0.92 |  | 0.54 |  | 1.56 |  |  |  | 0.76 |  |  |  |  |  |
|  | **Log** ∑**DMAP 2** |  | 0.80 |  | 0.39 |  | 1.67 |  |  |  | 0.56 |  |  |  |  |  |
| TRIMESTER 3 | **Log** ∑**DAP 3** |  | 1.01 |  | 0.65 |  | 1.57 |  |  |  | 0.96 |  |  |  |  |  |
|  | **Log** ∑**DEAP 3** |  | 0.99 |  | 0.62 |  | 1.60 |  |  |  | 0.98 |  |  |  |  |  |
|  | **Log** ∑**DMAP 3** |  | 1.05 |  | 0.72 |  | 1.51 |  |  |  | 0.81 |  |  |  |  |  |
|  |  |  |  |  |  |  |  |  |  |  |  |  |  |  |  |  |

| **Table S2c**: Covariate and creatinine adjusted^*^ odds ratios for PROFILE 3: Low attention, self-regulation and high excitability/arousal per inter-quartile increase in pesticide biomarkers, Study of Asian Women and their Offsprings’ Development and Environmental Exposure (2017-2019) |  |  |  |  |  |  |  |
| --- | --- | --- | --- | --- | --- | --- | --- |
|  |  |  | **Odds Ratio** | **95% Confidence Limits** |  | **p value** |  |
| OVERALL | **Log** ∑**DAP** |  | 0.55 | 0.34 | 0.88 | 0.012 |  |
|  | **Log** ∑**DEAP** |  | 0.45 | 0.24 | 0.85 | 0.013 |  |
|  | **Log** ∑**DMAP** |  | 0.99 | 0.95 | 1.02 | 0.41 |  |
| TRIMESTER 1 | **Log** ∑**DAP 1** |  | 0.45 | 0.24 | 0.85 | 0.013 |  |
|  | **Log** ∑**DEAP 1** |  | 0.50 | 0.28 | 0.89 | 0.019 |  |
|  | **Log**  ∑ **DMP 1** |  | 0.73 | 0.43 | 1.22 | 0.22 |  |
| TRIMESTER 2 | **Log** ∑**DAP 2** |  | 0.43 | 0.22 | 0.84 | 0.013 |  |
|  | **Log** ∑**DEAP 2** |  | 0.44 | 0.24 | 0.79 | 0.0065 |  |
|  | **Log** ∑**DMAP 2** |  | 0.80 | 0.37 | 1.75 | 0.58 |  |
| TRIMESTER 3 | **Log** ∑**DAP 3** |  | 0.72 | 0.44 | 1.19 | 0.20 |  |
|  | **Log** ∑**DEAP 3** |  | 0.65 | 0.39 | 1.09 | 0.10 |  |
|  | **Log** ∑**DMAP 3** |  | 0.99 | 0.66 | 1.47 | 0.94 |  |

^*^Covariates used in all models: father’s education, infant sex, amenities/appliances in home, having at least one previous preterm birth, gestational age, and maternal TONI-IV; creatinine included in models as an independent variable.
